# CRF2 Signaling Is a Novel Regulator of Cellular Adhesion and Migration in Colorectal Cancer Cells

**DOI:** 10.1371/journal.pone.0079335

**Published:** 2013-11-18

**Authors:** Benjamin Ducarouge, Marjolaine Pelissier-Rota, Michèle Lainé, Nadine Cristina, Yvan Vachez, Jean-Yves Scoazec, Bruno Bonaz, Muriel Jacquier-Sarlin

**Affiliations:** 1 Equipe du Stress et des Interactions Neurodigestives, INSERM U836, Grenoble, France; 2 Université Joseph Fourier, Grenoble Institut des Neurosciences, Grenoble, France; 3 Hôpital Edouard Herriot, Lyon, France; 4 Centre Hospitalo Universitaire de Grenoble, Grenoble, France; Kaohsiung Chang Gung Memorial Hospital, Taiwan

## Abstract

Stress has been proposed to be a tumor promoting factor through the secretion of specific neuromediators, such as Urocortin2 and 3 (Ucn2/3), however its role in colorectal cancer (CRC) remains elusive. We observed that Ucn2/3 and their receptor the Corticotropin Releasing Factor receptor 2 (CRF2) were up-regulated in high grade and poorly differentiated CRC. This suggests a role for CRF2 in the loss of cellular organization and tumor progression. Using HT-29 and SW620 cells, two CRC cell lines differing in their abilities to perform cell-cell contacts, we found that CRF2 signals through Src/ERK pathway to induce the alteration of cell-cell junctions and the shuttle of p120ctn and Kaiso in the nucleus. In HT-29 cells, this signaling pathway also leads to the remodeling of cell adhesion by i) the phosphorylation of Focal Adhesion Kinase and ii) a modification of actin cytoskeleton and focal adhesion complexes. These events stimulate cell migration and invasion. In conclusion, our findings indicate that CRF2 signaling controls cellular organization and may promote metastatic potential of human CRC cells through an epithelial-mesenchymal transition like process. This contributes to the comprehension of the tumor-promoting effects of stress molecules and designates Ucn2/3-CRF2 tandem as a target to prevent CRC progression and aggressiveness.

## Introduction

Colorectal cancer (CRC) is the second leading cause of cancer-related death in Western countries. Histological grade is an important prognostic marker as high-grade, poorly differentiated tumors are usually more aggressive and invasive than their low-grade, well-differentiated counterparts. A hallmark of CRC is loss of cellular organization. Adhesive interactions between cells and extracellular matrix (ECM) are key determinants of tissue organization and their modulations participate in cell migration and tumor metastasis.

In epithelial cells, cell-cell adhesion is maintained through several protein complexes such as adherens junctions (AJ). Cadherins are transmembrane proteins that nucleate AJ by forming homotypic calcium dependent interactions with cadherins from neighboring cells. Manipulation of E-cadherin function in the intestinal epithelium has revealed an important role in cell differentiation or cell/matrix adhesion [1]. E-cadherin is diminished in invasive CRC together with the acquisition of a mesenchymal phenotype [2]. The intracellular domain of E-cadherin interacts directly with β- and p120 catenins (ctn). They regulate AJ by controlling cadherin clustering, endocytosis or stability and actin cytoskeleton anchorage (reviewed in [3]). In E-cadherin deficient cells p120ctn shuttles to the cytoplasm and/or the nucleus where it exerts different functions depending on its partners [4], [5]. In the nucleus, p120ctn can interact with the transcription factor Kaiso and relieves its gene repression activity [6], [7]. Abnormal nuclear localization of p120ctn and Kaiso is prognostic for aggressiveness in CRC [8].

Micro-environment controls cancer progression through cell contacts or mediator signals [9]. The corticotropin releasing factor (CRF) and analogs like urocortins (Ucns) [10] are secreted peptides related to stress. They act through two G protein coupled receptors, CRF1 and CRF2, with different affinities [11]. CRF and Ucn1 bind both receptors, while Ucn2/3 are selective for CRF2. CRF receptors are primarily coupled to Gαs and trigger cAMP formation via adenylyl cyclase activation [12]. If CRF system is well documented in the gastrointestinal tract for its expression and regulation by stress and inflammation, its implication in CRC is poorly investigated [13], [14]. Ligands and receptors are expressed and secreted by various normal and cancer cells. Therefore, CRF system could modulate the tumor micro-environment by autocrine/paracrine activations on cancer or stromal cells [9], [15], [16]. The aim of this study was to determine the expression of CRF2 and its ligands in CRC and how their signaling could participate in the tumor progression. Our results described aberrant expression of CRF2 and ligands in both CRC tumors and cell lines, according to their grade and/or differentiation status. Using the HT-29 and SW620 cell lines, we discovered that CRF2 signaling modifies cellular adhesion and established a mechanism by which stress molecules may participate in tumor progression.

## Materials and Methods

### Cell culture

The human colon adenocarcinoma cell lines HT-29 and SW620 obtained from the American Type Culture Collection (ATCC, Manassas, VA) were cultured at 37°C in a 5% CO2 atmosphere in DMEM containing 25 mM glucose (Invitrogen, Cergy Pontoise, France) and supplemented with 10% FCS, 5% penicillin and streptomycin. The human CRF2-GFP construct was cloned into the pBabe expression vector. Retroviral infections of HT-29 cells were done as described previously [17] and then cultured in medium containing 2 µg/ml puromycin (BD Biosciences), following to FACS selection of virus-infected cells.

### Antibodies and reagent

Polyclonal antibodies directed against CRF2 were from Abcam (12964, Paris, France). The immunizing peptide used to generate the CRF2 antibody was designed in the conserved sequence of the α, β and γ isoforms. This antibody would then recognize all isoforms of the receptor. Anti-human E-cadherin (HECD1) monoclonal antibody was obtained from Takara Biochemicals (Cambrex Bio Science, Paris, France). Monoclonal antibody against p120ctn (clone 98) was purchased from BD Transduction Laboratories (Pont de Claix, France). Anti-actin, anti-MMP3 polyclonal antibodies, and anti-Kaiso, anti-vinculin monoclonal antibodies were obtained from Sigma Aldrich (L'Isle d'Abeau, France). Polyclonal antibodies directed against Src-P^Tyr418^ were purchased from MBL Calbiochem (Fontenay-sous-Bois, France), monoclonal anti-Src and anti-MMP7 antibodies were from Millipore (Molsheim, France). Monoclonal antibodies against ERK and ERK-P^Tyr204^ were from BD transduction laboratories and Santa cruz from TEBU (Le perray en Yvelines, France) respectively. Polyclonal anti-FAK-P^Ser910^ antobodies were obtained from Invitrogen (Cergy Pontoise, France). Alexa–conjugated goat anti-mouse secondary antibody was obtained from Molecular Probes (Eugene, OR). Horse Radish Peroxydase–conjugated goat anti-mouse secondary antibody was obtained from Bio-Rad (Marnes-la-Coquette, France), donkey anti-rabbit antibodies were obtained from Jackson Immunoresearch (Immunotech, Marseille, France). Topro3 was purchased from Invitrogen. Urocortins, CRF and Astressin 2b were purchased from Sigma Aldrich.

### Patient cohort, Tissue Micro Arrays and immunohistochemistry

This study was conducted on cDNA of a cohort of 30 CRC patients. Samples from frozen tumor and peritumoral tissues were obtained from the Tumor Tissue Bank of Hospices Civils de Lyon, supported by National Institute of Cancer (INCa) and French Ministery of Health. The tissue bank conforms to French regulations. All patients have given written informed consent to the use of tissue samples for research purposes; in the absence of informed consent, tissue samples from patients known to be deceased could also be used for research purposes, in accordance with French regulations. Procedures for collection, storage and release of tissue samples are in accordance with national and international recommendations and a quality management programme has been developed. All projects submitted to the tissue bank are reviewed and approved by its Scientific Committee, which also verifies their conformity to ethical regulations. To preserve anonymity, a specific ID was attributed to each patient. For each sample, the cDNA from tumoral tissue was paired with the cDNA of the adjacent normal tissue. The tumor stages were defined according to the TNM status of the tumors (American Joint Committee on Cancer Staging system). Tissue Micro Arrays (TMA) CDA3 were purchased at SUPER BIO CHIPS (Korea). The slide was blocked in TBS/BSA 3%/Tween-20 0.1% and incubated over night at 4°C with anti-CRF2 at 1∶100. The further IHC was performed with Vectastain ABC Kit (Vector Laboratories, Courtaboeuf, France), counterstained with 1 min incubation in Hematoxylin, dehydrated and mounted with DPX. Every microscope acquisitions were down in “TRIBUN” and quantified with Image J. The IHC quantifications were performed in ImageJ WCIF with the CIE Lab function of the color based thresholding segmentation to separate the CRF2 staining from the Hematoxylin counter-staining. Six different fields of every sample were segmented and the mean gray value was calculated for the CRF2 and the Hematoxylin. The CRF2 intensity for each sample is the mean of the CRF2/Hematoxylin ratio calculated on the six different fields.

### Cell fractionation

For nuclear fractionation, cell were lysed in HEPES 10 mM/MgCl2 1.5 mM/KCl 10 mM/DTT 0.5 mM/NP40 0.05%/pH 7.9. After a 10 min centrifugation at 3000 rpm and 4°C, pellets were resuspended in HEPES 5 mM/MgCl2 1.5 mM/EDTA 0.2 mM/DTT 0.5 mM/glycerol 26% (v/v)/pH 7.9, and homogenized with 20 full strokes in Dounce. After 30 min incubation on ice, lysates were centrifuged 20 min at 24000 g and 4°C. Supernatants containing nuclear extracts were analyzed by immunoblot.

### Immunoblot and Immunoprecipitation

Total lysates or subcellular fractions were processed for immunoblot as described previously [18].

### Immunofluorescent staining

Cells were grown on glass coverslips and then treated as described previously [18]. After fixation with PFA 4% sucrose, non-specific sites were blocked for 1 hr at 37°C with 3% BSA 0.5% Tween20. Then cells were incubated for 1 hr at 37°C with specific antibodies which were diluted in the blocking solution at 1∶100 for primary and 1∶500 for secondary antibodies. Fluorescence photomicrographs were taken with a confocal microscope at the ×100 objective (Leica TCS SPE) or an epifluorescence microscope at the ×100 objective (ZEISS, AvioVert 200M).

### RT and qPCR

Total RNA extractions were performed using Trizol^TM^ reagent and 1 µg of total RNA was denaturized and subsequently processed for reverse transcription using M-MLV (Invitrogen) according to manufacturer's instructions. qRT-PCR were done with a Light cycler 480 and the universal probe library (Roche). Primer sequences and probes are summarized in Table 1.

**Table 1:** Oligonucleotide sequences of primers used for qPCR and RT-PCR.

	qPCR	Forward	Reverse	UPL
**PGK**	X	ctgtggcttctggcatacct	cgagtgacagcctcagcata	**42**
**HPRT**	X	tgaccttgatttattttgcatacc	cgagcaagacgttcagtcct	**73**
**Ucn2**	X	tgccccacagagtcacagt	tgtggcagtgacccaactta	**64**
**Ucn3**	X	ggagggaagtccactctcg	gggcttggctttgtagaactt	**4**
**CRF2α**	X	tcctgcgaaatgtcatgtg	tgaagatggtggtgatgcag	**18**
**Cdh1**	X	tggaggaattcttgctttgc	cgctctcctccgaagaaac	**84**
**Krt20**	X	tgtcctgcaaattgataatgct	agacgtattcctctctcagtctcata	**66**
**Vim**	X	tggtctaacggtttccccta	gacctcggagcgagagtg	**56**
**MMP3**		tgtagaaggcacaatatgg	cagtcacttgtctgttgcaca	**-**
**MMP7**		atggggaactgctgacatc	ccagcgttcatcctcatcga	**-**
**GAPDH**		tcctcctgcacagtca	caccaccttcttgatgtcatc	**-**

GAPDH, glyceraldehydes-3-phosphate dehydrogenase; MMP, matrixmetalloproteinase; PGK, phosphoglycerate kinase; HPRT, human phosphoribosyltransferase.

### ECM preparation and cell adhesion

Tissue culture dishes were coated with LM-332 using the following methods: A431 epidermoid cells were cultured to confluence on various surfaces at 37°C to allow for the deposit of LM-332, then cells were removed as previously described [19], [20]. Briefly, confluent monolayers were sequentially extracted with 1% (v/v) Triton X-100 in PBS, followed by 2M urea in 1M NaCl. Plates were washed in PBS, incubated with 1%BSA, and stored at −20°C. Human collagen type IV from placenta was obtained from Sigma Aldrich. Coating of plastic Petri dishes (Microtiter plates 96-well, Nunclone; Nunc, Roskilde, Denmark) was performed by overnight incubation with ECM proteins (10 µg/mL) at 4°C. Plates were saturated with 3% (w/v) BSA in PBS for 2 hrs at 37°C. HT-29 CRF2-GFP cells were pretreated or not with 100 nM Ucn3 for 30 min before to be plated (5×10^4^ cells/well) in triplicate in coated 96-well microtiter plates and incubated from 0 to 60 min at 37°C. Non adherent cells were removed by washing three times with PBS, and cell adhesion was estimated by a cell proliferation assay (CellTiter 96 AQ_ueous_ Non-Radioactive Cell Proliferation Assay; Promega).

### Cell transmigration and invasion

HT-29 CRF2-GFP cells were seeded on 24 well transwells (8 µM pore size) (Becton Dickinson). After cell confluence, the culture medium was serum harvested over night in up and down chambers. Transmigration was then initiated by establishment of a serum gradient with 10% FCS in the lower chamber, with or without 100 nM Ucn3 in each chamber. 72 hrs after the initiation of transmigration, cotton swabs were used to remove cells on the upper surface of transwells. After fixation with PFA 4%, migratory cells were stained with Hematoxylin and manually counted under the microscope. The mean values +/− SEM were calculated and analyzed using Student's test.

HT-29 CRF2-GFP cells (2.5 10^−5^/mL) were placed in the top compartment of a 24-multiwell insert insert plate (BD Falcon) that was separated from the bottom compartment by BD-Matrigel Matrix membrane, with 0.4-µm pore size. Serum-free RPMI with or without Ucn3 (1 µM) were added into the top compartment and 10% FCS into the bottom compartment. After 48 hrs at 37°C in a 5% CO2 atmosphere, cells that had invaded through the Matrigel, were analyzed as described before for the transmigration assay.

Cell viability was evaluated with the CellTiter 96 Kit (Promega) according to the manufacturer's instructions.

### Gelatin degradation assay

About 40 µL (20-30 µg cell protein) harvested culture medium were electrophoresed under nonreducing conditions in a 10% acrylamide gel containing 1 mg/mL gelatine (Sigma-Aldrich), according to the method described by Werb and al., [21]. After electrophoresis, the gels were washed at room temperature for 3×30 min in 2% Triton X-100 and overnight at 37°C in buffer containing 20 MM Tris-HCl (pH 7.7) and 5 mM CaCl_2_.Thereafter, gels were stained with 0.1% (wt/vol) Coomassie Brillant Blue R-250 in 50% (vol/vol) isopropyl alcohol/10% (vol/vol) glacial acetic acid for 60 min and destained in 20% (vol/vol) isopropyl alcohol/5% (vol/vol) glacial acetic acid.

### Densitometric analysis and statistics

Immunoblots shown are representative of at least three independent experiments. All graphs represent the mean value ± SD of protein expression levels measured by densitometric analysis in “Image J” software (NIH). The relative expression levels of mRNA or proteins were determined as follows: the expression of level of each transcript or protein was normalized to i) their housekeeping gene in qPCR or RT-PCR; ii) to actin in whole lysate westernblots or histones in nuclear extract westernblots. In some experiments and in order to display a fold increase over control, the relative expression of transcripts or proteins in control conditions was set to 1. Statistics are unpaired t-test and statistical significance is given by the number of asterisks (*P<0.05; **P<0.01; ***P<0.001).

## Results

### Expression of CRF2 and its ligands increase with staging in CRC tissues and cell lines

**Figure 1:**
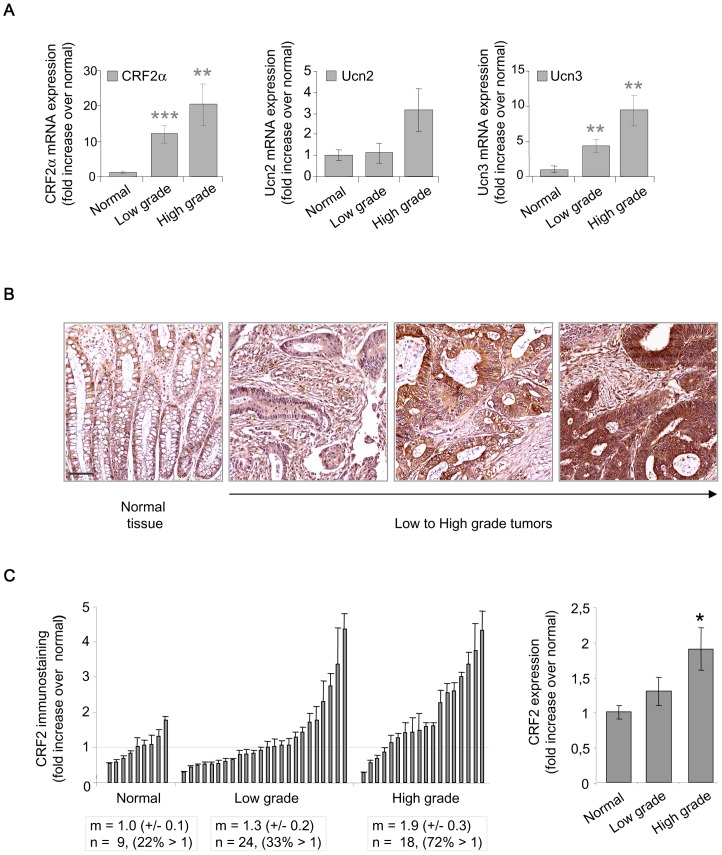
CRF2 and ligand expression are increased in high grade and poorly differentiated human CRC. (A) Histograms representing CRF2α, Ucn2 and Ucn3 transcript expression normalized to the housekeeping gene PGK (phosphoglycerate kinase) in human normal tissues or CRC (low and high grades). (B) Representative CRF2 immunohistochemistry performed on CRC tissues from low to high grade. Scale bar, 50 µm. (C) Histograms representing CRF2 protein expression quantified from immunohistochemistry. Each sample is represented as dark bars +/− SD (left) and the mean value of each group +/− SD (right). High grade is statistically different from normal at *, p<0.05.

**Figure 2:**
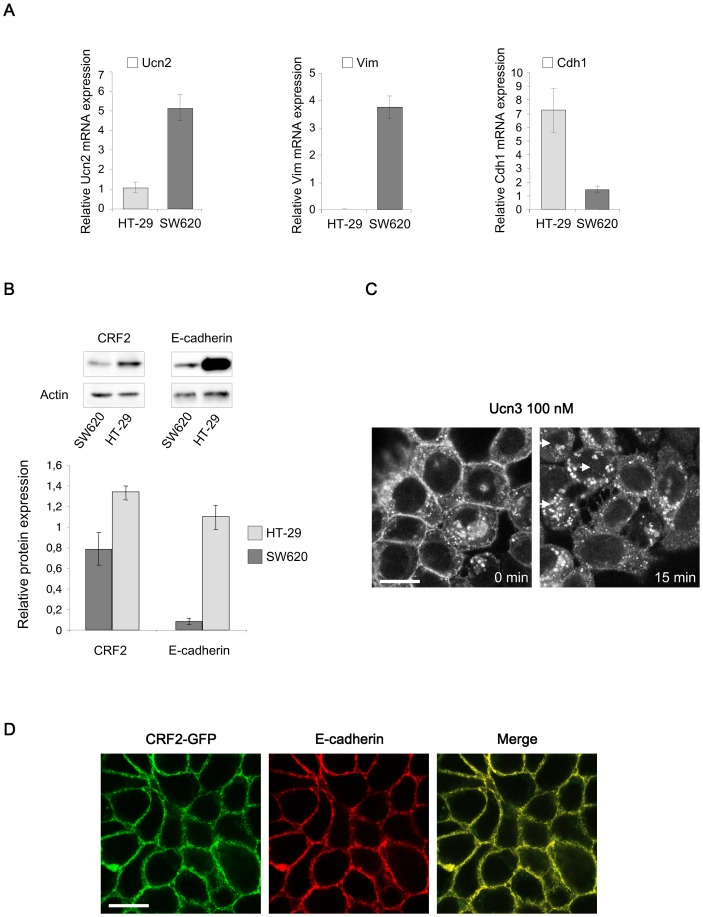
Characterization of CRF2/Ucns and cell differentiation marker expression in CRC cell lines. (A) Characterization of HT-29 and SW620 cell lines by mRNA expression of Ucn2, Vimentin (Vim) and E-cadherin (Cdh1) normalized to the housekeeping gene HPRT (human phosphoribosyltransferase). (B) E-cadherin/actin and CRF2/actin protein expression quantified by immunoblot in SW620 and HT-29 cell lines. (C) Confocal microscopy analysis of CRF2-GFP distribution in Ucn3-treated HT-29 cells. Scale bar, 15 µm. (D) Confocal microscopy analysis of CRF2-GFP and E-cadherin distribution in HT-29 cells. Scale bar, 20 µm.

We have first performed qPCR for CRF2α (the major CRF2 isoform of colonic epithelium), [22] and Ucn2/3 in normal and CRC tissues of 30 patients, to better understand their regulation during tumor progression (Figure 1A). The clinicopathological findings of the tumors used in the study are shown in Table 2. Tumors were clustered in low (n = 19, corresponding to stages 1–2) and high (n = 11, corresponding to stages 3–4) grades and standardized to their normal tissues. Statistical analysis revealed a significant increase of CRF2α and Ucn3 mRNA expression in tumors compared to normal tissues according to the tumor grade. There were no significant differences of Ucn2 transcripts in these conditions. However, when the analysis was done in tumors tissues only, a pattern was revealed regarding Ucn2 mRNA expression: Ucn2 transcript levels were increased according to the pNM status, the lymph node invasion and microsatellite instability (MSI). Finally, CRF2α, Ucn2 and Ucn3 transcript levels didn't correlate to mutations of *K-ras or B-raf*. A similar result was found with CRC cell lines (supplemental Figure S1). Immunohistochemical analysis was used in order to detect CRF2 receptor protein expression in colorectal sections corresponding to normal tissues (n = 9), low grade (n = 24) and high-grade (n = 18) tumors (Figure 1B). The proportion of samples greater than the mean of normal tissues increased from 22% in normal to 33% and 72% in low and high-grade tumors respectively (Figure 1C, left panel). Moreover, the CRF2 protein expression increased according to the tumor grade (ANOVA p = 0.047) (right panel). These results suggest a relationship between CRF2 protein expression and higher tumor grades. We extended our analysis by qPCR in 17 human CRC cell lines (supplemental Figure S1). Interestingly, we found that the expression of CRF2 ligands is inversely correlated to epithelial differentiation markers like E-cadherin (*Cdh1*) but correlated to mesenchymal makers like vimentin (*Vim*), suggesting a role of CRF2 in the cellular disorganization observed during tumor progression. Two CRC cell lines were selected: i) HT-29 cells, which express high level of E-cadherin but low level of Ucn2 and no vimentin; ii) SW620 cells, poorly differentiated mesenchymal cells which express high levels of Ucn2 and vimentin compared to a weak level of E-cadherin (Figure 2A). The expression of CRF2 and E-cadherin at the protein level was confirmed by immunoblotting (Figure 2B). Using HT-29 cells that stably overexpress the CRF2 coupled to GFP protein (HT -29 CRF2-GFP cells) we observed that CRF2-GFP was predominantly localized at the membrane, in intercellular contacts (Figure 2C). This observation was further supported by microscopy confocal analysis showing a co-localization of CRF2-GFP protein with E-cadherin in HT-29 cells (Figure 2D). With Ucn3 (the most efficient CRF2 agonist in HT-29 cells, see supplemental Figure S2), cells contacts were altered and CRF2-GFP shifted in cytoplasmic vesicles (Figure 2C).

**Table 2:** Patient's clinicopathological findings.

Age	Median 75 (16–92)										
Sex	F	n = 12									
	M	n = 18									
Localization	Left colon	n = 13									
	Right colon	n = 17									
mRNA study		Cases	CRF2α			Ucn2			Ucn3		
Characteristics		(n = 30)	Mean	SEM	pvalue	Mean	SEM	pvalue	Mean	SEM	pvalue
pTNM	pN0M0	19	12	2,4	0,539	1,1	0,5	**0,038**	4,3	0,9	0,165
	pN1M0	4	28,1	13,8		0,5	0,3		7,8	2,5	
	pN0M1	3	13,4	5,1		5	2,5		11,5	5,7	
	pN1M1	4	18	9,2		4,4	1,5		9,6	4,4	
Lymph node	N0	23	14,8	3,2	0,787	1	0,4	**0,005**	4,9	0,9	0,082
	N1	7	16	5,4		4,7	1,3		10,4	3,2	
Metastasis	M0	22	12,2	2,1	0,26	1,6	0,6	0,527	5,3	1,2	0,205
	M1	8	23	7,9		2,4	1		8,7	2,4	
Grade	Normal	30	1	0,4	**<0,001**	1	0,3	0,157	1	0,4	**<0,001**
	Low	19	12	2,4		1,1	0,5		4,3	0,9	
	High	11	20,4	5,9		3,2	1		9,5	2,2	
Kras/B-Raf	WT	12	14,7	3,4	0,982	2,6	1	0,127	7,7	1,9	0,177
	Mut	17	16,3	4,1		1,2	0,5		4,9	1,2	
MSI	MSS	24	15,1	3,2	0,237	1,2	0,4	**0,019**	5,1	1	0,094
	MSI	5	18,1	4		4,5	2		10,4	3,6	

Altogether, these data indicate that CRF2 and its ligands are expressed in human CRC and cell lines according to the tumor grade and/or differentiation status. CRC cells could activate their CRF2 via an autocrine production of ligands, especially in undifferentiated cell lines.

### Regulation of cell-cell contacts by CRF2 signaling

**Figure 3:**
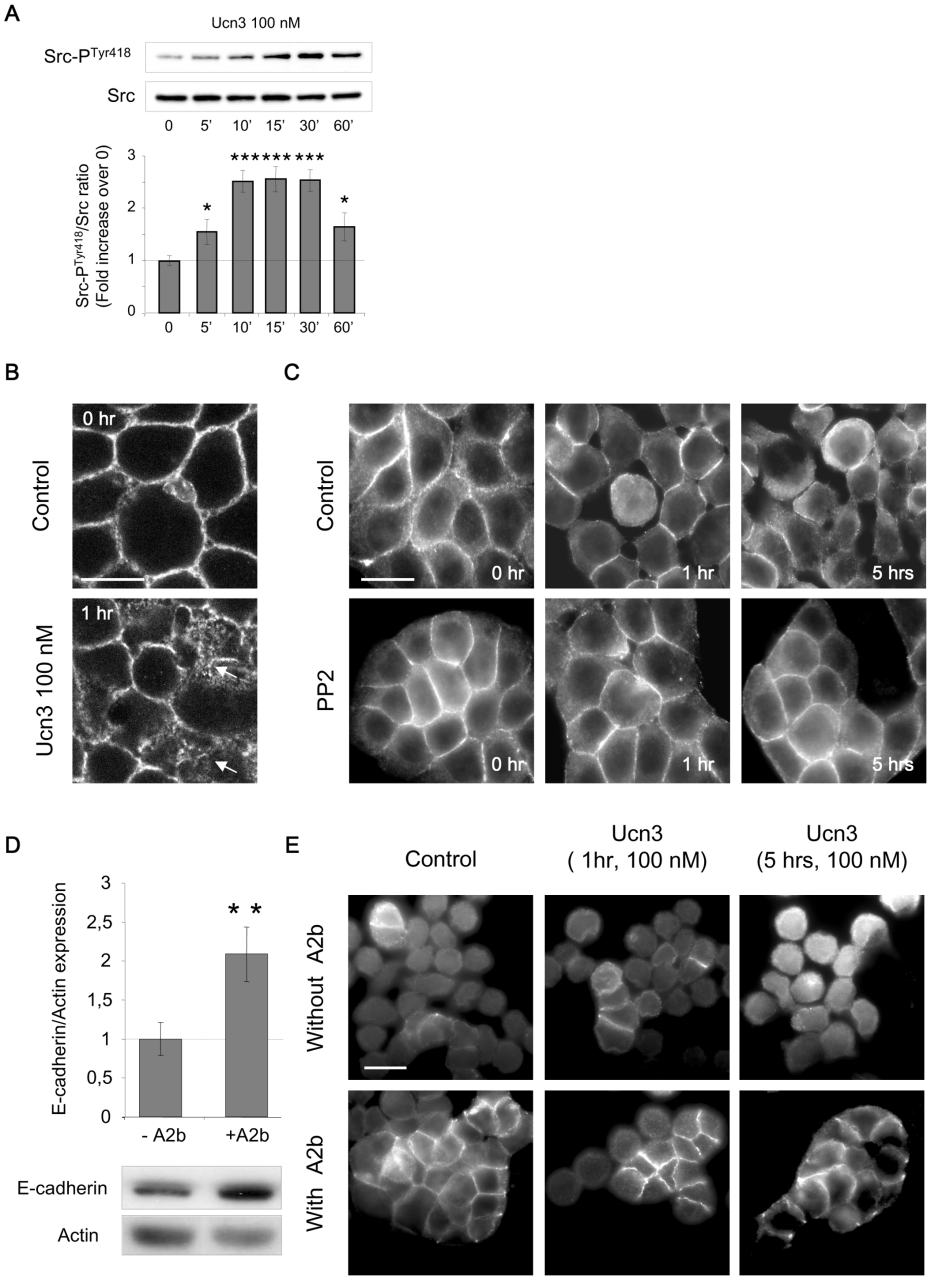
CRF2 signaling disrupts AJ. (A) Immunoblot analysis of Src-P^Tyr418^ and Src total expression in HT-29 cells following the time course of Ucn3 treatment. (B) Confocal analysis of E-cadherin distribution in HT-29 cells pretreated with cycloheximide before Ucn3 treatment. Scale bar, 15 µm. (C) Effect of PP2 (10 µM, 1 hr) on E-cadherin distribution in HT-29 cells treated or not with Ucn3, analyzed by epifluorescence microscopy. Scale bar, 20 µM. (D) Effect of CRF2 antagonist (A2b, 8 nM, 24 hrs) on E-cadherin expression in SW620 cells. Quantification was performed from immunoblots and normalized to actin. (E) E-cadherin distribution in SW620 cells pretreated or not with A2b and further treated or not with Ucn3. Analyzed were performed by epifluorescence microscopy. Scale bar, 10 µM.

CRF2 signalization has been recently extended to non canonical G PROTEIN COUPLED RECEPTOR pathways, including Src kinase which directly interacts with the endocytosed CRF receptors and takes part in the activation of ERK [23], [24]. Src-P^Tyr418^ expression was progressively increased from 5 to 30 min, while the total Src remained unchanged (Figure 3A). The phosphorylated form of Src has been co-immunoprecipitated with CRF2 during the early stage of the kinetic (supplemental Figure S3). In CRC, Src kinase activation correlates to E-cadherin disturbance, histological grading and poor prognosis [25]. We thus investigated the involvement of Src activity in Ucn3-mediated cell dissociation. In HT-29 cells, confocal microscopy analysis showed that E-cadherin staining produced a honeycomb like pattern at the lateral membrane cortex (Figure 3B). Activation of CRF2 by Ucn3 rapidly altered this pattern. Only a thickened and disrupted linear membrane staining persisted while numerous cytoplasmic accumulations appeared, indicating an endocytosis of E-cadherin associated to AJ disruption. Cycloheximide was used to prevent the accumulation of neosynthesized E-cadherin. Internalization assays using HECD-1 antibodies were done to confirm the Ucn3-induced E-cadherin endocytosis (Figure supplemental S4). Cell dissociation and endocytosis of E-cadherin, upon Ucn3 exposure was prevented by pre-treatment with the Src family inhibitor PP2, indicating that Src activation was required for the Ucn3-mediated cell-cell contacts disruption (Figure 3C). In SW620 cells, which express low level of E-cadherin and perform few AJ, blockade of CRF2 signaling by its specific agonist astressin2b (A2b) for 24 h was responsible of an increased expression of E-cadherin (Figure 3D). Immunofluorescence analysis of E-cadherin distribution in SW620 cells showed weak membrane expression and cell-cell contacts with or without Ucn3 (Figure 3E). In presence of A2b, SW620 cells formed clusters and E-cadherin was localized at the cell-cell contacts. Ucn3 slightly reversed A2b-induced cell clustering. Modulation of adhesive proprieties of SW620 cells were also evidenced in aggregation assays, showing that both adherent and non adherent cells were able to aggregate in presence of A2b (supplemental Figure S5).

**Figure 4:**
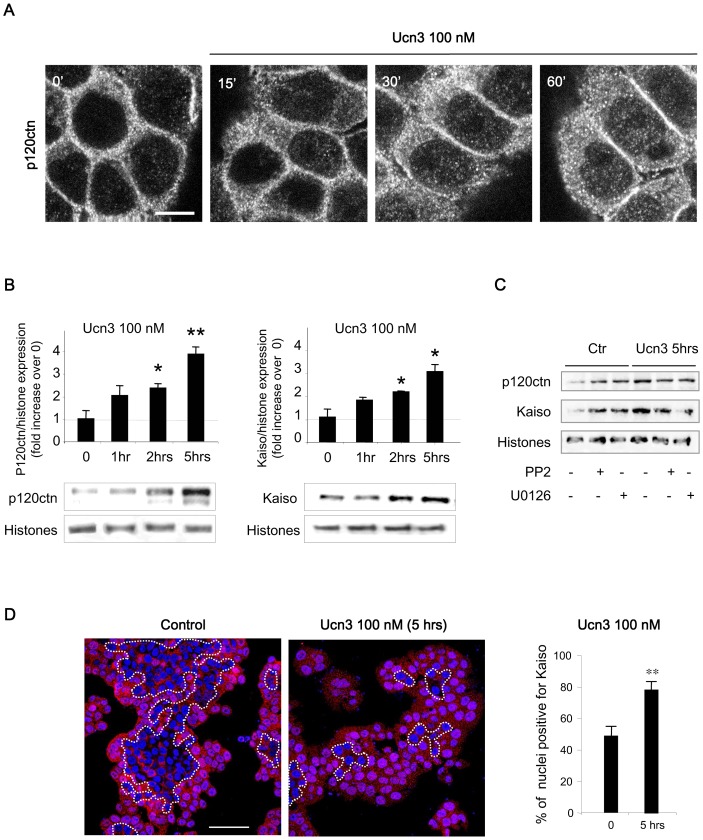
CRF2-mediated dissociation of AJ leads to nuclear shuttling of p120ctn and Kaiso. (A) Confocal analysis of p120ctn location in HT-29 cells pretreated with cycloheximide before Ucn3 treatment. Scale bar, 10 µm. (B) Immunoblot analysis of p120ctn and Kaiso protein expression in nuclear extracts from HT-29 cells treated or not with Ucn3 (expression was normalized to histones). (C) Effects of Src and MEK inhibitors (PP2 and U0126: 10 µM, 1 hr) on p120ctn and Kaiso protein expression in nuclear extracts from HT-29 treated or not with Ucn3. Expression was normalized to histones. (D) Confocal analysis of cellular distribution of Kaiso in HT-29 cells. Nuclei were stained with Topro3. Cells negative for nuclei labeling of Kaiso were circumscribed by a white dotted line. Scale bar, 60 µm.

E-cadherin is associated with catenins at AJ complexes. Disruption of cell-cell contacts leads to AJ proteins dissociation and cytoplasmic and/or nuclear localization of catenins, which promote cell invasion in CRC [26]. In HT-29 cells treated with Ucn3, p120ctn was accumulated in the cytoplasm and the nucleus (Figure 4A). In the nucleus, p120ctn has been described to regulate the activity of the transcription factor Kaiso. Ucn3 significantly increased p120ctn and Kaiso nuclear expression as observed by immunoblotting after nuclear fractionation (Figure 4B). To identify the signaling molecules involved in this nuclear shuttling, we tested the effect of Src (PP2) and MEK (U0126) inhibitors. As shown on Figure 4C, we found that these inhibitors reversed the Ucn3-induced nuclear accumulation of these proteins. They also induced an increase of basal nuclear expression of p120ctn and Kaiso, probably due to their toxic effect. The nuclear distribution of Kaiso was further analyzed by confocal microscopy (Figure 4D). The percentage of nuclei positive for Kaiso increased about 40% in HT-29 cells treated with Ucn3. Interestingly, in control cells, cells corresponding to nuclei positive for Kaiso were observed at the periphery of the cluster, which corresponds to cells with less intercellular contacts. Under Ucn3, cells at the centre of the cluster became positive for nuclei labeling of kaiso.

Taken together our data indicate that CRF2 signals through a Src pathway to induce AJ disruption by E-cadherin endocytosis. This is followed by p120ctn and Kaiso nuclear translocation. Through this route, CRF2 may participate in cell dedifferentiation and migration.

### CRF2 signaling reorganizes cell-matrix contacts in favor of cell migration and invasion

**Figure 5:**
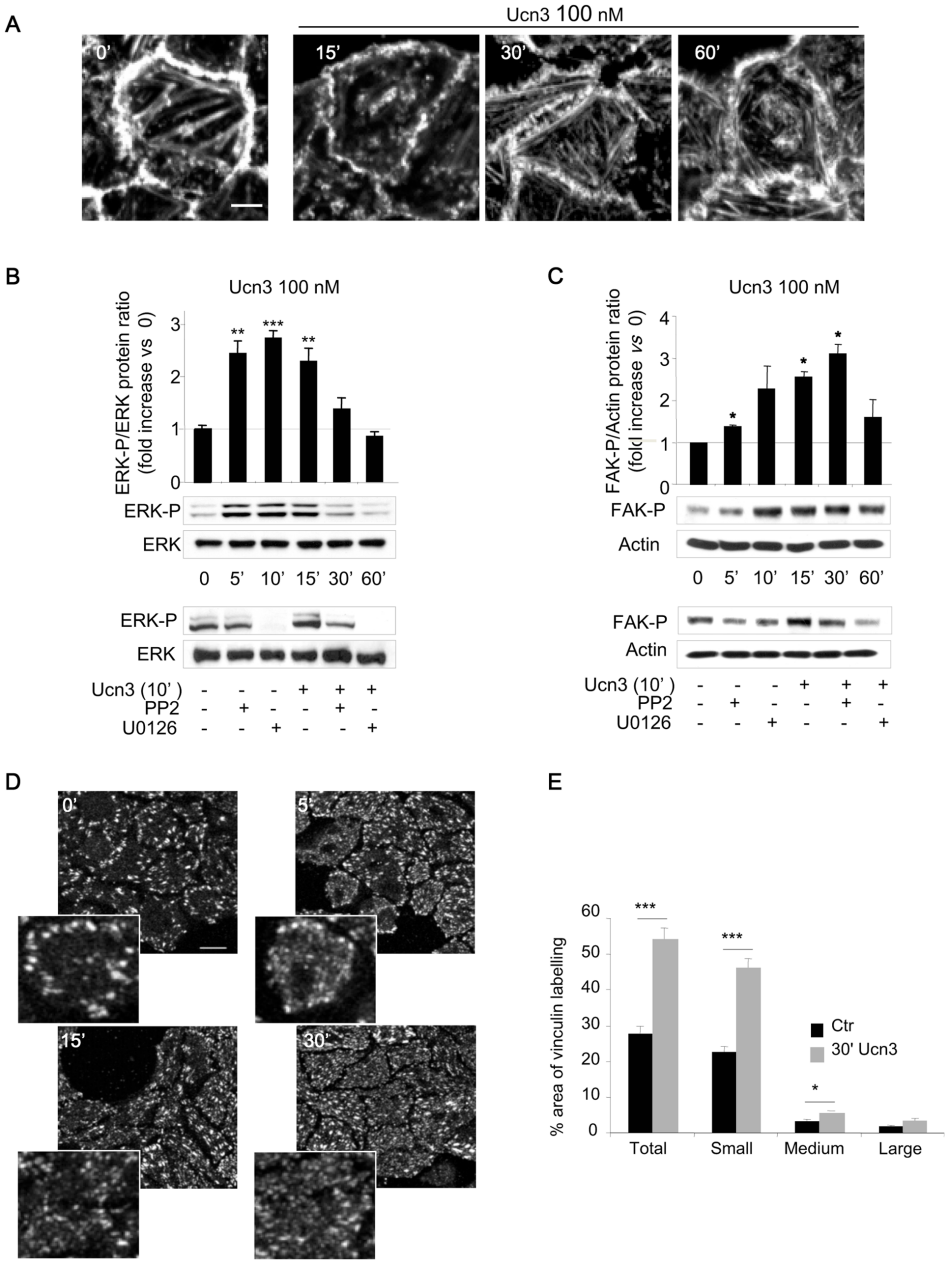
CRF2 signaling reorganizes cell-matrix contacts. (A) Phaloidin-TRITC fluorescence is analyzed by confocal microscopy at the basal pole of HT-29 CRF2-GFP cells following Ucn3 treatment. Scale bar, 5 µm. (B) ERK-P^Tyr204^/ERK total ratio quantified from immunoblot in HT-29 cells treated with Ucn3 (0 to 60 min) (Upper panel). Effects of Src (PP2: 10 µM, 1 hr) or MEK (U0126: 10 µM, 1 hr) inhibitors on Ucn3 induced ERK-P^Tyr204^ (Lower panel). (C) Ucn3-mediated phosphorylation of FAK- P^Ser910^ in HT-29. Quantifications were performed from immunoblots and normalized to actin (upper panel). Effects of Src (PP2, 10 µM, 1 hr) and ERK (U0126, 10 µM, 1 hr) inhibitors on Ucn3 induced FAK-P^Ser910^ (Lower panel). (D) Confocal analysis of vinculin distribution at the basal pole of HT-29 following Ucn3 treatment. Scale bar, 20 µm. (E) Quantification of the patch number by size at 30 min of Ucn3 treatment.

Cancer cell migration is required for metastasis and results from the reorganization of cell-cell and cell-matrix contacts. Ucn3-induced cell dissociation was also associated with cell shape remodeling as observed by actin cytoskeleton staining with phalloidin-TRITC at the basal pole of HT-29 cells (Figure 5A). In untreated cells, actin was distributed at the cell periphery and organized as stress fibers spread across the cells. With Ucn3, the actin staining was less intense at the cell periphery where contacts are dissociated. There were also fewer stress fibers, which appeared shorter and more fragmented. The remodeling of cell shape is driven by intra-cellular kinases signaling. In this line, the ERK pathway has also often been activated in response to CRF2 activation. In HT-29 CRF2-GFP cells, we showed that ERK was phosphorylated on Tyr^204^ in a short time frame after Ucn3 exposure (Figure 5B). The ratio of phosphorylated/total ERK was increased by a 2.5 fold between 5 and 15 min and returned to its basal level at 30 and 60 min. PP2, reversed the ERK activation induced by Ucn3 without changing its basal level (low panel). The MEK inhibitor, U0126, induced the loss of the phosphorylated forms of ERK under basal conditions and after Ucn3 treatment. Cell adhesion remodeling and migration involves the focal adhesion (FA) kinase (FAK). This kinase could be phosphorylated on Ser^910^ by the ERK Ser/Thr kinase. We have thus determined the level of FAK phosphorylation in HT-29 CRF2-GFP cells exposed to Ucn3 (Figure 5C). FAK P^Ser910^ was transiently increased with a maximum at 30 min. This phosphorylation was slightly inhibited by PP2 and U0126 under basal conditions and abolished under Ucn3 treatment. These data suggest that Ucn3 activates a Src/ERK/FAK phosphorylation cascade through the CRF2 receptor.

**Figure 6:**
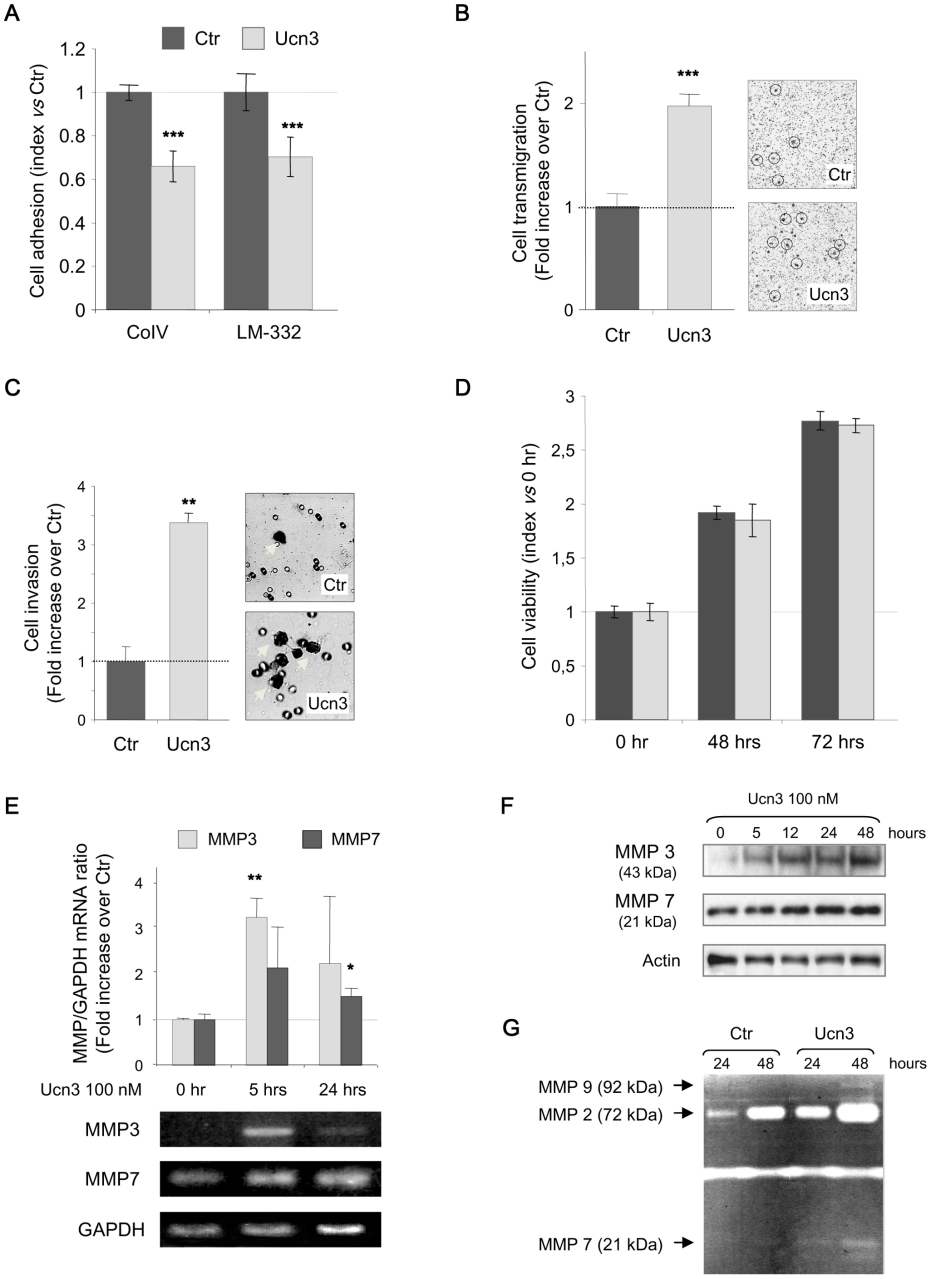
CRF2 signaling alters cell adhesion in favor to cell migration and invasion. (A) HT-29 were treated or not with Ucn3 (100 nM) 30 min before adhesion and then plated on either COIV or LN-332. Cell-ECM adhesion was evaluated at 60 min with the use of a Cell Titer Aqueous MTT reagent kit. Values are normalized to the adhesion without Ucn3. Data represent the mean +/− SEN of 4 separate experiments. Effect of Ucn3 on HT-29 cells transmigration after 72 hrs was determined using a transwell assay (B) and on HT-29 cell invasion after 48 hrs by a matrigel assay (C). In both assays cells were visualized by phase contrast microscopy and counted. A phase contrast image has been joined. A circle has indicated some migrating cells and some invaded cells have been indicated by a white arrow. Data (n>3) are given as mean +/− SEM. (D) Effects of Ucn3 on HT-29 cell viability. (E) Regulation of MMP3 and MMP7 mRNA expression analyzed by RT-PCR in HT-29 cells treated with Ucn3 100 nM. (F) Immunoblot analysis of MMP3 and MMP7 protein expression in HT-29 cells following the time course of Ucn3 treatment. (G) Zymogram of gelatinolytic activities in supernatant of HT-29 cells treated or not with Ucn3 (100 nM) during 24 and 48 hrs.

Focal adhesion structures, which are regulated by FAK, participate in cell-ECM interactions. These interactions are mediated by various transmembrane receptors such as integrins, linked to cytoskeleton components and scaffold molecules like vinculin [27]. We analyzed the effect of Ucn3 on FA through the distribution of vinculin (Figure 5D). Without Ucn3, vinculin was expressed in strong patches distributed mainly at the cell periphery. With Ucn3, smaller punctuated claws appeared in vinculin staining that were homogeneously distributed all over the cell and still not restricted at the periphery, especially at 30 min. The quantification of these patches by size revealed that small patches increased while large remain unchanged (Figure 5E). This label suggests the formation of nascent focal contacts, which have been previously described to participate in cell adhesion and migration [28]. We have performed cell adhesion experiments in order to test if the Ucn3-induced vinculin reorganization could alter cell attachment to collagen IV (CoIV) and laminin 332 (LM-332) (two major ECM proteins of colon's basal lamina) (Figure 6A). Ucn3 decreases HT-29 cell adhesion on these two matrix components at 20 and 60 min of plating. To determine if this effect was associated to migration and/or to invasion we tested the impact of Ucn3 on HT-29 cell motility by transwell migration and Matrigel invasion assays (Figures 6B and C). Ucn3 treatment significantly increased the level of cell migration and invasion compared to control cells. This effect was not supported by modification of cell viability (Figure 6D), but could be regulated by secretion of matrix metalloproteases (MMPs). We hence examined the expression and the activity of MMPs. As shown in Figure 6E, mRNA levels for MMP3 and MMP7 were found to be transiently increased by Ucn3. We didn't observed significant modulation of MMP2 and MMP9 transcripts (data not shown). These results were confirmed at protein levels (Figure 6F). We next examined by a gelatin degradation assay, if HT-29 cells secreted functionally active MMPs that have been reported to play a role in carcinoma invasion through extracellular matrix degradation. As shown in Figure 6G, MMP enzyme activity was present in unstimulated cell line supernatants, and stimulation with Ucn3 resulted in an increase in MMP activity.

Altogether, these data indicate that CRF2 signaling induced important cell adhesion remodeling. At the basal pole, ECM and cell-matrix contacts are remodeled, leading to a decreased adhesion on CoIV and LM-332 substratum. These modifications favor cell migration and invasion and provide evidence that CRF2 signaling may contribute to invasive phenotype of CRC.

## Discussion

The CRF2/Ucns system is up-regulated in high grade CRC. Our *in vitro* data highlight the functional relevance of this CRF2 over-signaling in the regulation of cell adhesion that contribute to epithelial-mesenchymal transition (EMT)-like process with enhance migration and invasion.

CRF receptors and ligands are expressed in many types of cancers and cancer cell lines [29], [30]. Their inappropriate secretion is sometimes correlated with tumor aggressiveness [31]. However no value as tumor marker has been found for CRF receptors in lung and breast cancer respectively, whereas in endometrial cancer, CRF1 expression is correlated with less aggressive tumors, whereas CRF2 expression is increased in the cytoplasm of advanced stage tumor cells [32]. In the colon, we found that CRF2 expression (at transcript and protein levels) was increased in CRC according to their grade and/or differentiation status. Likewise Ucn2/3 are overproduced in high-grade tumors and there is a balance between Ucn2 expression and epithelial markers observed in CRC cell lines suggesting that an autocrine activation of CRF2 could take part in the progression of CRC cells.

Thus it is clear that the two CRF receptors exhibit different distributions (cellular and subcellular) and hold distinct roles in cancer cells, which could even be counteracting. CRF signaling, in particular CRF1, has been described to regulate either tumor initiation and progression or tumor inhibition, affecting cell proliferation, apoptosis or tumor angiogenesis (for review [15], [33]) while CRF2 may play a role in the invasiveness [16], [34]. In this work, we first described that CRF2 may also contribute to an EMT-induced cell disorganization and dedifferentiation that could be associate to metastatic progression. Indeed, in HT-29 cells, we found that CRF2 activation induced weakness and disruption of AJ, a process associated to the endocytosis of E-cadherin expression and to the nuclear localization of p120ctn and Kaiso. Inversely, in SW620 cells, which express low level of E-cadherin, blockade of CRF2 autocrine activation by A2b induces E-cadherin re-expression and cell clustering. Src kinase activity is increased in many CRC and has been described to trigger cell-cell junction disassembly [35] and induce nuclear translocation of p120ctn in tumor cells lacking E-cadherin [5], [36]. An association between Src and CRF1 following short-term treatment with Ucn has been initially described in cardiomyocytes and plays an essential role in urocortin-mediated cardioprotection [23]. We observed that Src is rapidly activated (phosphorylation on tyr418) and recruited to CRF2 in response to Ucn3 signaling. Pretreatment with PP2 abolished Ucn3-induced disruption of cell-cell contacts and p120ctn/Kaiso nuclear translocation suggesting an active role of Src in these effects. P120ctn nuclear translocation could relieve Kaiso-mediated repression of several cancer-related genes, such as MMP7 or Wnt11 (for review [7]). In addition to its repression activity, Kaiso also contains enhancer motifs in which the function of p120ctn binding is unknown [37]. We found that Ucn3 induced both the regulation of p120ctn/Kaiso nuclear ratio and the transcription of MMP3 and MMP7. These results were confirmed at protein levels. Ucn3 also induced a secretion of MMP2 and MMP9 in cultured medium measured by zymography. However MMP2 and MMP9 mRNA expression was unaffected by Ucn3 under the conditions of our experiments, indicating that Ucn3 may also regulate MMP production at the level of posttranslational processing. A similar regulation of MMP9 by Ucn has been described in cultured cells from human placenta [38]. During cancer progression, these MMP enhance cell migration and invasion by degrading ECM components [39] or extracellular fragment of E-cadherin, thus disrupting AJ [40].

Elevated nuclear levels of Kaiso are frequently seen in human cancers including CRC and Kaiso-deficient mice show resistance to intestinal cancer [41]. Interestingly, invasive cells at the border of the tumor have increased levels of nuclear Kaiso [42]. In HT-29 cells, cells positive for nuclear kaiso were principally found at the periphery of the cell cluster. Under Ucn3, positive cells for nuclear kaiso reached the center of cell cluster. The nuclear localization of kaiso that correlates to reduction of contacts with the cell matrix or surrounding cells could represent an indicator of cell adhesion dynamic. Our *in vitro* assays establish conditions that activate colon cancer cell motility through a Src/ERK/FAK pathway, thus supporting a role for CRF2 signaling in tumor progression and metastasis. These observations would need to be supported by *in vivo* assays. In CRC, transient ERK activation seems to be sufficient to induce FAK phosphorylation on Ser^910^ and subsequent migration and metastasis [43], [44]. In HT-29 cells, the CRF2 is also responsible for a transient increase in ERK activation that leads to FAK-P^Ser910^. Furthermore, activated Src is required to activate ERK, since PP2 also abolished Ucn3-induced phosphorylation of ERK. This signaling could modulate the association of FAK with the scaffold protein paxillin [45], which regulates the FA turnover and actin cytoskeleton plasticity at the basal pole of the cell. Structurally, we found that CRF2 activation leads to a decrease of organized stress fibers in favor of shorter and less polarized ones. This cytoskeleton reorganization occurs concomitantly with the decrease of cell adhesion on ECM and the formation of small vinculin patches. This is in agreement with smaller patches of paxillin and vinculin observed in metastatic-derived cell lines (SW620) compared to their primary tumor-derived cell lines (SW480) [28]. Previous studies have demonstrated that the alteration of FA complexes is involved in the transmigration of SW480 and HT-29 cells [46]. The CRF2-induced impairment of AJ combined to FA complexes remodeling could favor HT-29 cell transmigration.

Stress participates in the development and aggravation of gastrointestinal disorders (for review [47], [48]). Indeed CRF2 and its ligands are overexpressed in the colon of inflammatory bowel diseases patients while AJ protein expression is altered [49]. We found that similar CRF2-dependent cell adhesion alterations occur in tumor cells thus providing a new role of stress-related CRF system in CRC. Overall, our data suggest that Ucn3 stimulates cell motility and invasiveness of HT-29 cells most probably *via* induction of FAK phosphorylation and actin filament reorganization. Based on these findings we postulate that the stress neuropeptide Ucn3 present in the vicinity of tumors (either produced locally by the tumor cells themselves or by nearby normal cells or secreted from the innervations of surrounding tissues) could promote an EMT-like process and play an important role on colon cancer cell metastatic capacity, providing a potential link between stress and tumor progression. According to its potential implication in growth, angiogenesis, migration and metastasis, *via* activation of locally expressed ligands, the CRF2 could be clinically exploited as a target for new therapeutic approaches.

## Supporting Information

Figure S1 **CRF2 expression is inversely correlated to cell differentiation markers in CRC cell lines.** Correlated mRNA expression of Ucn2 with Vimentin (Vim), E-cadherin (Cdh1) and Keratin 20 (Krt20) in 17 CRC cell lines, normalized to the house keeping gene HPRT (human phosphoribosyltransferase).
(TIF)

Figure S2 **Adenylyl cyclase-dependent production of cAMP in HT-29 cells.** cAMP production +/− SD by HT-29 cells treated with 1 µM CRF, Ucn2 or Ucn3 in presence of 1 µM of astressin2b (A2b) (gray bars) or not (dark bars). At the concentration of 1 µM, CRF, Ucn2 and Ucn3 induced respectively 0.5, 1.0 and 2.4 pM of cAMP/10^5^ cells, which was completely reversed by A2b, a selective CRF2 antagonist. *Method*: Cells were treated as described; lyzed in 0.1 M HCl, centrifuged (600 g 20 min at RT) and supernatant were quantified for cAMP using the ELISA kit: Correlate^TM^ EIA (Assay designs) according to the manufacturer's instructions. All experiments were done in triplicates.
(TIF)

Figure S3 **Interaction between SrcP^Tyr418^ and CRF2 receptor.** (A). The association between Src-P^Tyr418^ and CRF2 has been tested by co-immunoprecipitation experiments CRF2 was immunoprecipitated from total lysate of Ucn3-treated HT-29 cells and levels of Src-P^Tyr418^ and CRF2 were detected by immunoblots. (B) Confocal analysis of CRF2-GFP (green) and Src-P^Tyr418^ (red) distribution in HT-29 CRF2-GFP treated with 100 nM Ucn3 (left). Scale bar, 10 µM. Quantification of CRF2-GFP and Src-P^Tyr418^ co-localization (right). * are statistics vs t = 0 and $ are statistics versus 5 min of Ucn3. CRF2/Src-P^Tyr418^ interaction was increased after 5 and 10 min of exposure to Ucn3. Furthermore, confocal microscopy analysis indicated a 6-fold increase (at 5 min) in the % of CRF2-GFP co-localized with Src-P^Tyr418^ at the basal pole of HT-29 CRF2-GFP cells in presence of Ucn3. *Methods*: For immunoprecipitation experiments cells were lysed in RIPA buffer containing protease and phosphatase inhibitor cocktails. An equal volume of each condition was immunoprecipitated using the PureProteome^TM^ Protein A and Protein G Magnetic beads kit (Millipore) according to manufacturer's instructions. Mouse and Rabbit Trueblot antibodies used as secondary antibodies in immunoprecipitation experiments were from eBioscience (Cliniscience, Montrouge, France).
(TIF)

Figure S4 **Ucn3-induced E-cadherin endocytosis.** A) Confocal analysis of E-cadherin endocytosis. HECD-1 antibodies directed against the extra-cellular domain of the E-cadherin in HT-29 CRF2-GFP treated (right) or not (left) with Ucn3 100 nM. Scale bar, 5 µm. B) Westernblot analysis of E-cadherin and p120ctn according to a time course of Ucn3 (100 nM). *Methods*: Coverslips were incubated at 4°C with the HECD-1 antibody diluted at 1∶100 in PBS. After 1 h, unspecific binding was removed with three PBS washes and coverslips were returned to the initial cell culture condition, with or without Ucn3. As mentioned, cells were washed on ice with PBS/NaCl 0.5M/Acetic acid 0.5M/Azide 10 mM to remove antibodies associated with extracellular cadherins. Coverslips were then treated like immunofluorescence.
(TIF)

Figure S5 **Blockade of CRF2 signaling induces cell aggregation.** Effect of A2b (1 µM, Overnight) on cell clustering in culture conditions (left) and on cell aggregation assays (right). *Method*: HT-29 cells were harvested from monolayer cultures as previously described by (Nakagawa and Takeichi, 1995). To preserve the integrity of E-cadherins at the cell surface, cells were resuspended at a density of 10^6^ cells/ml of TBS containing 10 mM HEPES/1 mM CaCl2. Aggregation assays were performed in a 24-well plate saturated with BSA and extensively washed. The cell suspensions were incubated at 37°C in a gyratory shaker at 75 rpm for 30 min. Cell aggregation was observed with an inverted microscope (Zeiss Axiovert 135) under phase contrast and photographs were taken with a CCD camera (Hitachi Denshi, Ltd.). *Nakagawa, S., and Takeichi, M. (1995). Neural crest cell-cell adhesion controlled by sequential and subpopulation-specific expression of novel cadherins. Development* ***121*** *, 1321–32.*
(TIF)
