# Supplementary figures and images for: CRF2 Signaling Is a Novel Regulator of Cellular Adhesion and Migration in Colorectal Cancer Cells
Source: PLoS One. 2013 Nov 18;8(11):e79335. doi: 10.1371/journal.pone.0079335 (PMC3832608; doi:10.1371/journal.pone.0079335)

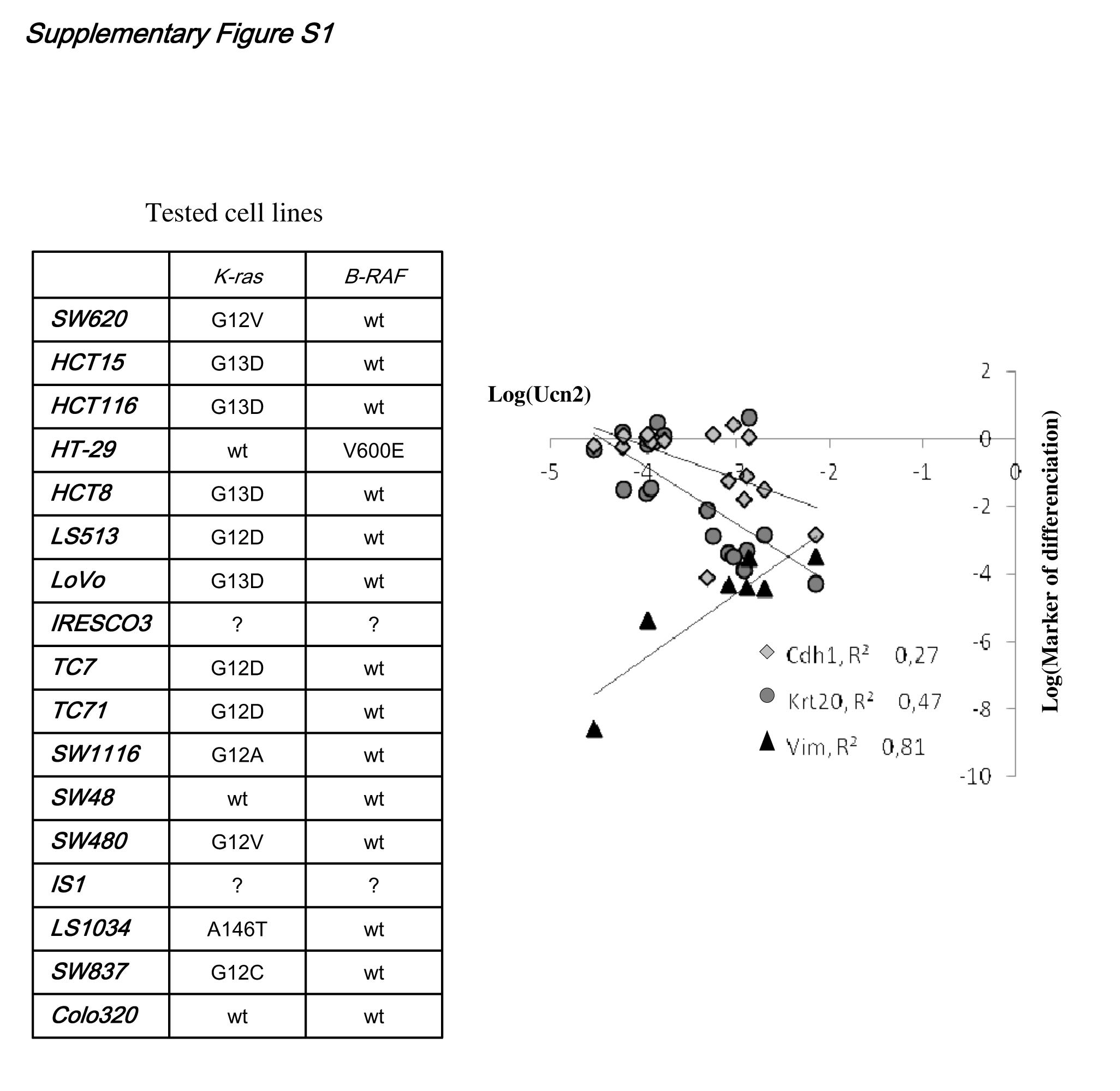

Supplement: Figure S1 — CRF2 expression is inversely correlated to cell differentiation markers in CRC cell lines. Correlated mRNA expression of Ucn2 with Vimentin (Vim), E-cadherin (Cdh1) and Keratin 20 (Krt20) in 17 CRC cell lines, normalized to the house keeping gene HPRT (human phosphoribosyltransferase). (TIF) [file pone.0079335.s001.tif]

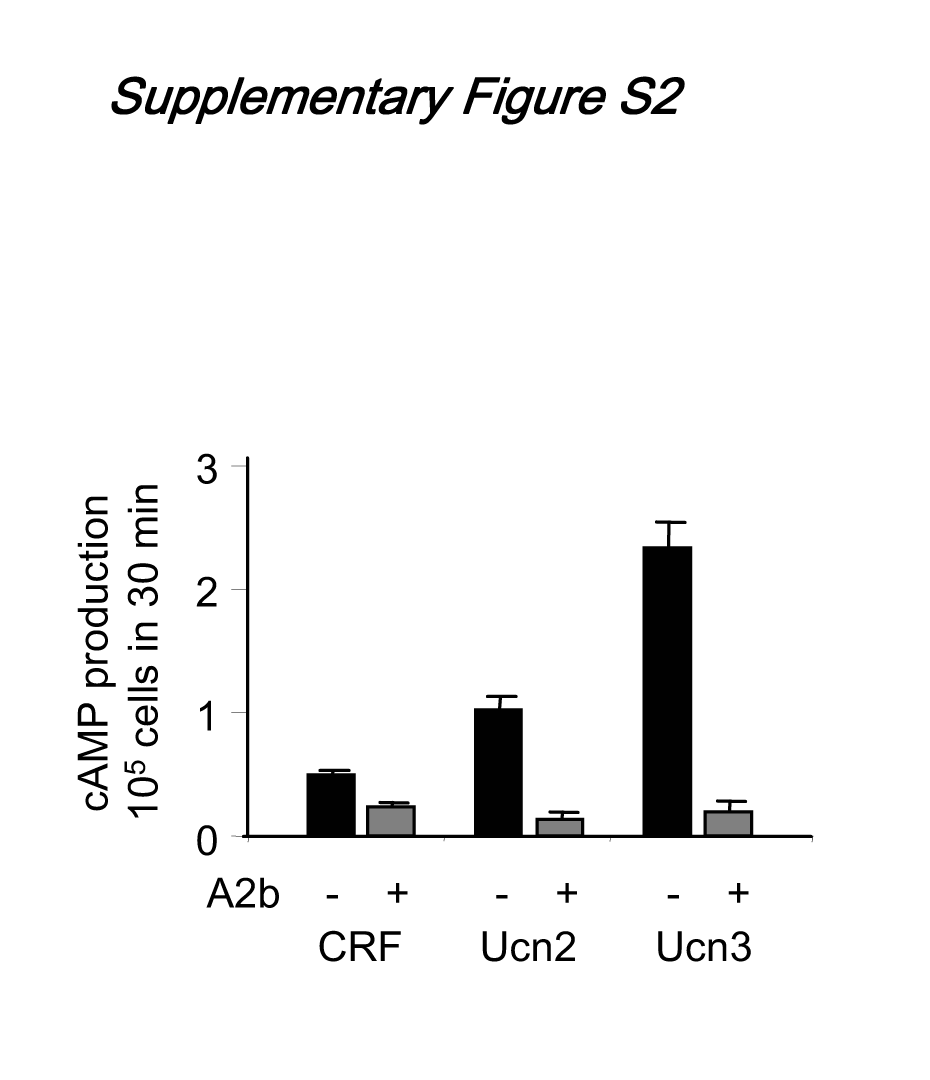

Supplement: Figure S2 — Adenylyl cyclase-dependent production of cAMP in HT-29 cells. cAMP production +/− SD by HT-29 cells treated with 1 µM CRF, Ucn2 or Ucn3 in presence of 1 µM of astressin2b (A2b) (gray bars) or not (dark bars). At the concentration of 1 µM, CRF, Ucn2 and Ucn3 induced respectively 0.5, 1.0 and 2.4 pM of cAMP/105 cells, which was completely reversed by A2b, a selective CRF2 antagonist. Method: Cells were treated as described; lyzed in 0.1 M HCl, centrifuged (600 g 20 min at RT) and supernatant were quantified for cAMP using the ELISA kit: CorrelateTM EIA (Assay designs) according to the manufacturer's instructions. All experiments were done in triplicates. (TIF) [file pone.0079335.s002.tif]

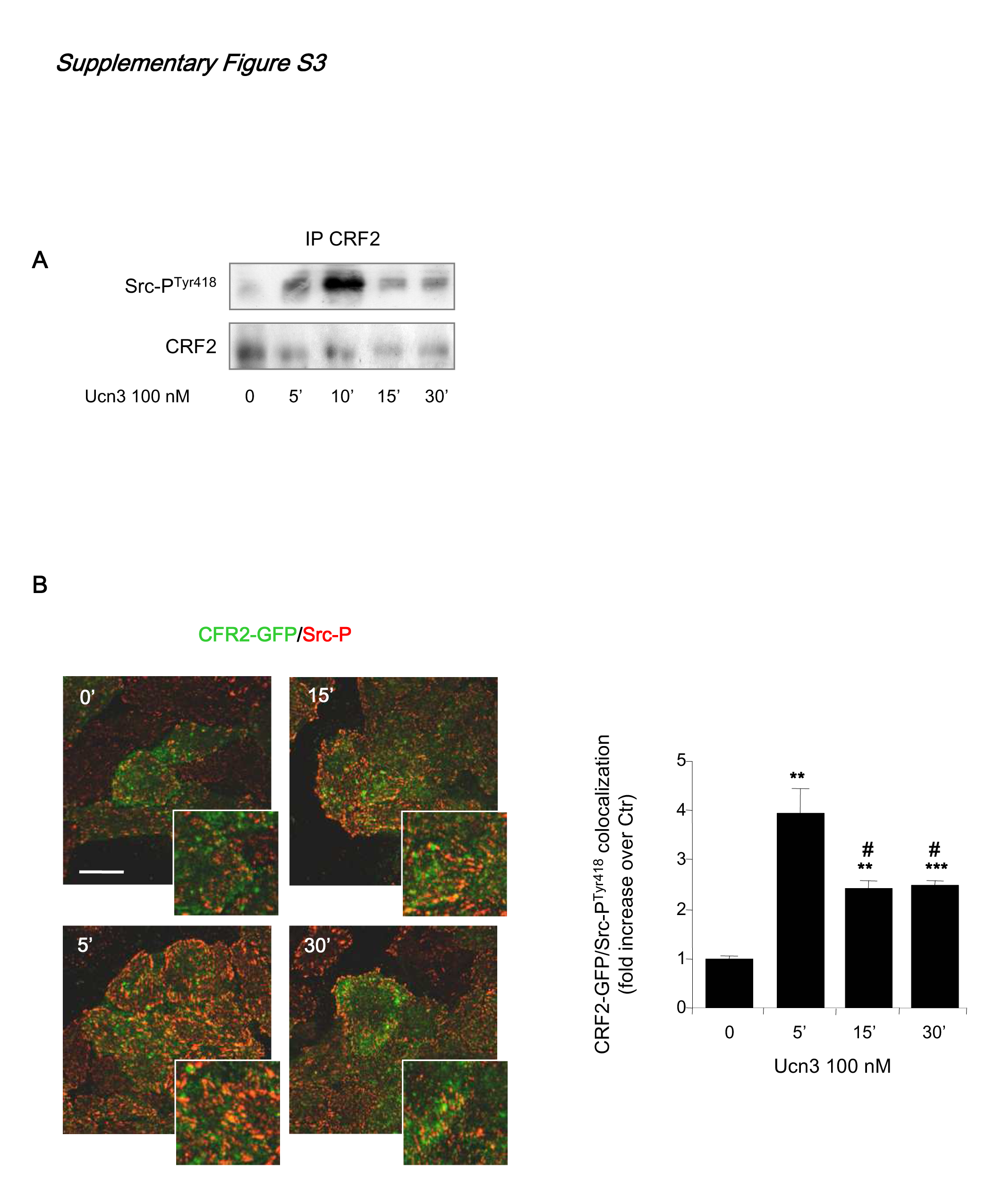

Supplement: Figure S3 — Interaction between SrcPTyr418 and CRF2 receptor. (A). The association between Src-PTyr418 and CRF2 has been tested by co-immunoprecipitation experiments CRF2 was immunoprecipitated from total lysate of Ucn3-treated HT-29 cells and levels of Src-PTyr418 and CRF2 were detected by immunoblots. (B) Confocal analysis of CRF2-GFP (green) and Src-PTyr418 (red) distribution in HT-29 CRF2-GFP treated with 100 nM Ucn3 (left). Scale bar, 10 µM. Quantification of CRF2-GFP and Src-PTyr418 co-localization (right). * are statistics vs t = 0 and $ are statistics versus 5 min of Ucn3. CRF2/Src-PTyr418 interaction was increased after 5 and 10 min of exposure to Ucn3. Furthermore, confocal microscopy analysis indicated a 6-fold increase (at 5 min) in the % of CRF2-GFP co-localized with Src-PTyr418 at the basal pole of HT-29 CRF2-GFP cells in presence of Ucn3. Methods: For immunoprecipitation experiments cells were lysed in RIPA buffer containing protease and phosphatase inhibitor cocktails. An equal volume of each condition was immunoprecipitated using the PureProteomeTM Protein A and Protein G Magnetic beads kit (Millipore) according to manufacturer's instructions. Mouse and Rabbit Trueblot antibodies used as secondary antibodies in immunoprecipitation experiments were from eBioscience (Cliniscience, Montrouge, France). (TIF) [file pone.0079335.s003.tif]

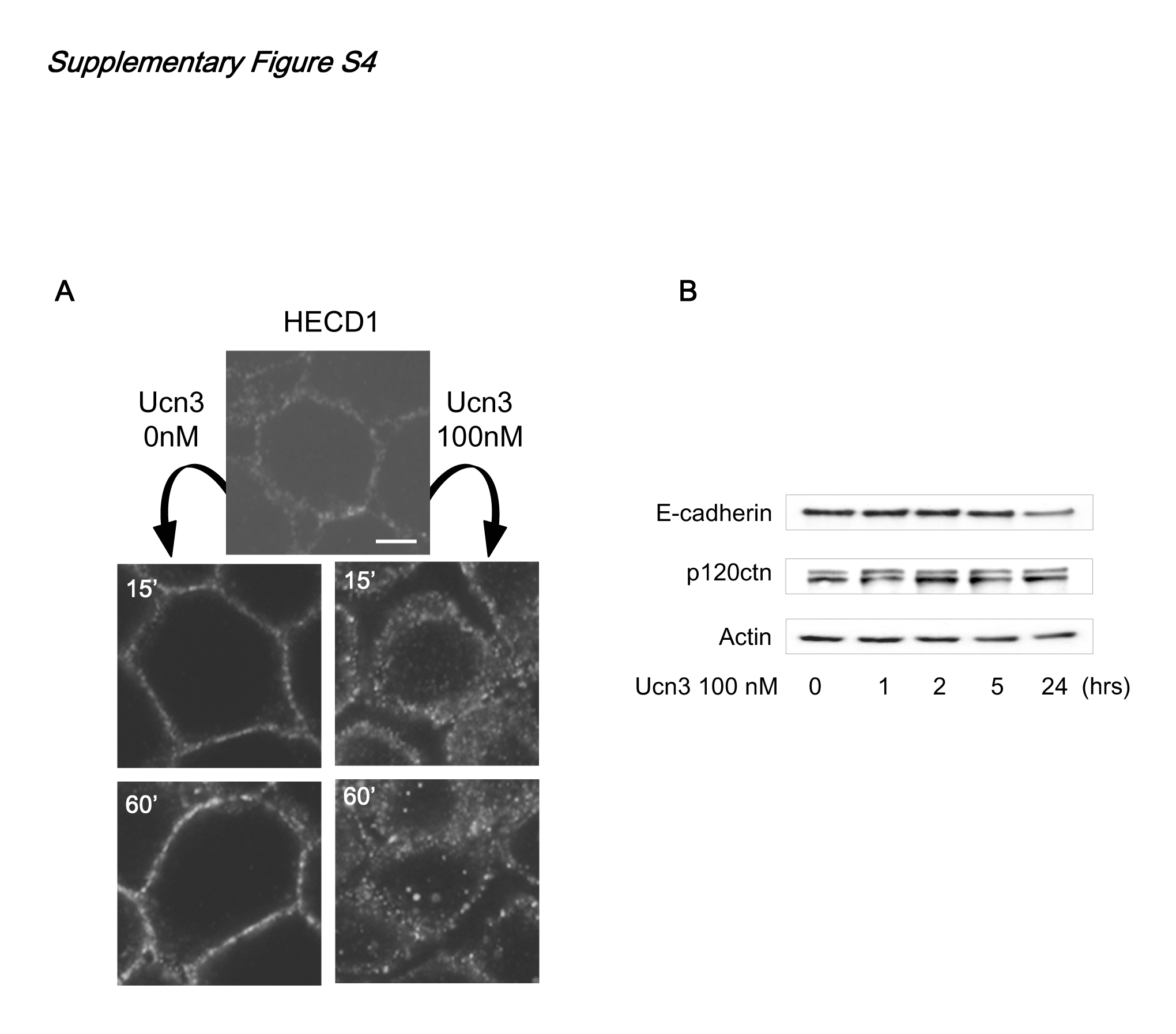

Supplement: Figure S4 — Ucn3-induced E-cadherin endocytosis. A) Confocal analysis of E-cadherin endocytosis. HECD-1 antibodies directed against the extra-cellular domain of the E-cadherin in HT-29 CRF2-GFP treated (right) or not (left) with Ucn3 100 nM. Scale bar, 5 µm. B) Westernblot analysis of E-cadherin and p120ctn according to a time course of Ucn3 (100 nM). Methods: Coverslips were incubated at 4°C with the HECD-1 antibody diluted at 1∶100 in PBS. After 1 h, unspecific binding was removed with three PBS washes and coverslips were returned to the initial cell culture condition, with or without Ucn3. As mentioned, cells were washed on ice with PBS/NaCl 0.5M/Acetic acid 0.5M/Azide 10 mM to remove antibodies associated with extracellular cadherins. Coverslips were then treated like immunofluorescence. (TIF) [file pone.0079335.s004.tif]

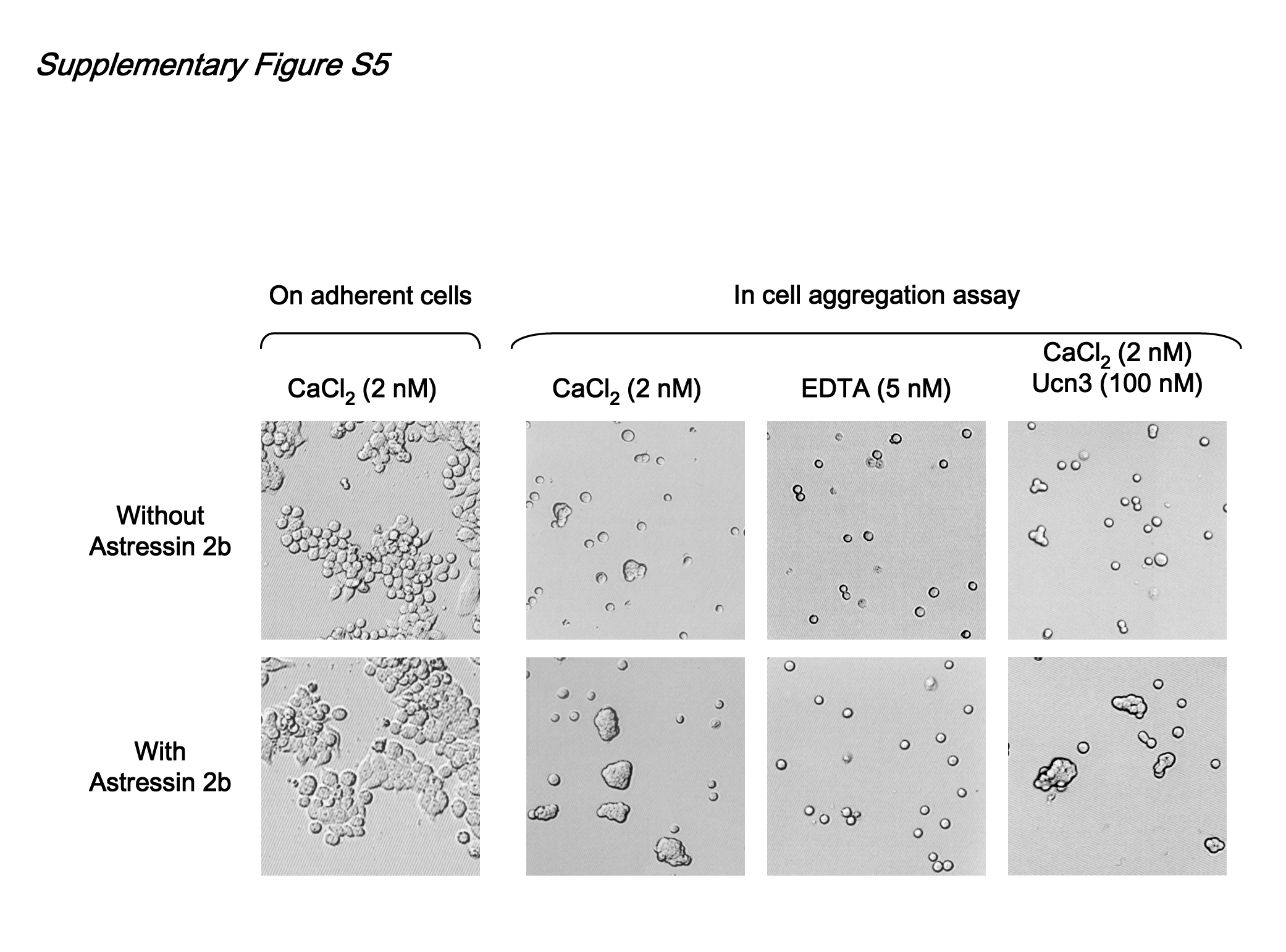

Supplement: Figure S5 — Blockade of CRF2 signaling induces cell aggregation. Effect of A2b (1 µM, Overnight) on cell clustering in culture conditions (left) and on cell aggregation assays (right). Method: HT-29 cells were harvested from monolayer cultures as previously described by (Nakagawa and Takeichi, 1995). To preserve the integrity of E-cadherins at the cell surface, cells were resuspended at a density of 106 cells/ml of TBS containing 10 mM HEPES/1 mM CaCl2. Aggregation assays were performed in a 24-well plate saturated with BSA and extensively washed. The cell suspensions were incubated at 37°C in a gyratory shaker at 75 rpm for 30 min. Cell aggregation was observed with an inverted microscope (Zeiss Axiovert 135) under phase contrast and photographs were taken with a CCD camera (Hitachi Denshi, Ltd.). Nakagawa, S., and Takeichi, M. (1995). Neural crest cell-cell adhesion controlled by sequential and subpopulation-specific expression of novel cadherins. Development 121 , 1321–32. (TIF) [file pone.0079335.s005.tif]
